# How Does Counselling in a Stationary Health Care Setting Affect the Attendance in a Standardised Sports Club Programme? Process Evaluation of a Quasi-Experimental Study

**DOI:** 10.3390/ijerph15010134

**Published:** 2018-01-14

**Authors:** Sylvia Titze, Christian Lackinger, Lena Grossschaedl, Albert Strehn, Thomas E. Dorner, Josef Niebauer, Wolfgang Schebesch-Ruf

**Affiliations:** 1 Institute of Sport Science, University of Graz, Mozartgasse 14, 8010 Graz, Austria; wolfgang.ruf@uni-graz.at; 2 Department of Health Promotion and Prevention, SPORTUNION Österreich, Falkestrasse 1, 1010 Vienna, Austria; c.lackinger@sportunion.at; 3 Social Insurance Authority for Business, Regional Office Styria, Körblergasse 115, 8010 Graz, Austria; lena.grossschaedl@svagw.at; 4 Competence Center Health Promotion, Social Insurance Authority for Business, Osterwiese 2, 7000 Eisenstadt, Austria; albert.strehn@svagw.at; 5 Department of Social and Preventive Medicine, Centre for Public Health, Medical University of Vienna, Kinderspitalgasse 15/1, 1090 Vienna, Austria; thomas.dorner@meduniwien.ac.at; 6 Institute of Sports Medicine, Prevention and Rehabilitation and Research Institute of Molecular Sports and Rehabilitation Medicine, Paracelsus Medical University Salzburg, Lindhofstrasse 20, 5020 Salzburg, Austria; j.niebauer@salk.at

**Keywords:** adults, attendance, health care setting, sports club, PRECIS-2

## Abstract

Actions in partnership across sectors is one principle for the promotion of health behaviours. The objective of this study was to describe the participation in a sports club-based exercise programme—named JACKPOT—following an intervention in a health care setting. Focus was given to the recruitment into JACKPOT, the attendance level, and whether the different programme elements were implemented as intented. The practicability of the project was also retrospectively rated. Participants were 238 inactive people (50% women) between 30 and 65 years of age who attended a health resort. Of these, 77% were assigned to the intervention group (IG). The recruitment into the 12 JACKPOT sessions and the attendance levels were recorded via attendance lists. The implementation of the intervention standards was assessed with structured interviews and participatory observation. The Pragmatic Explanatory Continuum Indicator Summary (PRECIS)-2 tool served to rate the practicability of the project. Almost 50% of the IG subjects attended JACKPOT sessions at least once and 54% of the attenders visited ≥75% of the 12 sessions. Some of the programme elements were not delivered fully. The process evaluation results showed that the project worked in a real-world setting, and also uncovered potential reasons such as incomplete information delivery for the moderate recruitment and attendance level.

## 1. Introduction

Regular and continuous physical activity (PA) has been shown to be beneficial for many cardiovascular risk factors [1,2,3]. However, the results of a population-based PA survey—as part of the Austrian Health Interview Survey (AT-HIS) in 2014—showed that only about a quarter (men 28%, women 22%) of the Austrian adult population meet the WHO [4] minimum requirements for health-enhancing PA, i.e., at least 150 min of moderate-intensity aerobic physical activity throughout the week and muscle-strengthening activities on 2 or more days a week [5]. Diverse sectors such as transport, education, urban design, health, and sports have been identified as potential drivers in the promotion of PA on a population level [6].

A recent publication by the U.S. Preventive Services Task Force [7] concluded that counselling interventions result in improvements in health-enhancing behaviour and small but potentially important improvements in intermediate outcomes among adults without obesity and other cardiovascular disease (CVD) risk factors.

In Austria, lifestyle counselling is one type of intervention provided during a residential stay in a health resort. A health resort is defined as an establishment that is specialised in the prevention of diseases and in tailored therapy through health education and active engagement of the people [8]. Adults with risk factors for cardio-metabolic or musculoskeletal system diseases who are also covered by an Austrian social insurance scheme can apply for a residential stay in a health resort twice over a five-year period, with the stipulation that the application has to be approved by the attending physician. In Styria, a federal state of Austria, approximately 16,000 of 598,832 adults in the age range of 30 to 65 years were assigned to attend a residential stay in a health resort in 2015 [9,10]. Stays last up to three weeks and are financed by social insurance companies. During the residential stay, the focus of both lectures and practical sessions is on PA, nutrition, and mental health. This health-promoting strategy has been faced with questions about how people keep up the newly adopted health behaviours because of the lack of—in the case of PA—regional health-enhancing PA programmes. 

The continuation of the new health behaviour, however, is a primary determinant of treatment success. In the scientific literature, the terms “attendance” and “adherence” are defined inconsistently. Based on Hawley-Hague et al. [11] and Visek et al. [12], the attendance level is an indicator—expressed, for example, as a percentage—of the number of sessions completed over a set period. In contrast, adherence informs us whether someone still attends sessions after the set period.

For intervention studies, the recruitment into health promotion programmes is also of major interest because it reflects whether the participant overcomes any pertinent barriers and is sufficiently motivated to engage in the intervention at least once [13].

Two basic approaches can be taken when designing an intervention study: a pragmatic trial or an explanatory trial [14]. A pragmatic trial is undertaken in the usual care settings, or “real world”. Explanatory trials test whether an intervention can have a beneficial effect under ideal conditions [15]. This project was designed with the intention to be pragmatic. Zwarenstein et al. [16] defined a pragmatic approach as a study design matched to the context, in which the intervention would be usable and the results applicable. The Pragmatic Explanatory Continuum Indicator Summary (PRECIS)-2 is a tool used to assess the practicability of a trial design. The outcome of the assessment tool helps health system decision-makers to answer the question as to whether the intervention works in the intended context [16,17]. 

To the best of our knowledge, only a limited number of studies have investigated the effectiveness of PA counselling in a health care setting on the adoption of PA in sports club programmes thereafter. Lackinger et al. [18] investigated the attendance level to a standardised programme (either gym-based or aqua-fit) covering two sessions per week among patients suffering from non-communicable diseases such as obesity, type 2 diabetes mellitus, or hypertension. General practitioners, outpatient departments, and health resorts recruited the participants. Among the 47 study participants who participated in the exercise programme for two months (=16 sessions), 29.8% were good attenders (>75 of all sessions), 23.4% attended between 51 and 75% of all sessions, and the others attended ≤50% of the sessions.

Based on the Medical Research Council guidance, the value of process evaluation is to learn about contextual factors associated with the outcome, the mechanism of impact (participants’ responses), and the intervention implementation (fidelity, dose, and reach) [19]. Thus, the main objective of the current study was to describe the participation in a standardised sports club-based exercise programme following PA counselling in a stationary health care setting. In particular, the aim was to investigate: (1) the proportion of study participants successfully recruited; (2) how consistently people attended the 12 sports club based sessions; and (3) whether members of the health resorts and exercise instructors implemented the different programme elements as intended. We also retrospectively rated the practicality of the project by applying the PRECIS-2 tool including domains such as organization and delivery of intervention or follow-up measurement.

## 2. Materials and Methods

### 2.1. Study Overview

The project with the incorporated standardised sports-club-based exercise programme, named JACKPOT, is a quasi-experimental study that has been previously described in-depth [20]. The study is registered at ClinicalTrials.gov (identifier: NCT02552134). The protocol was approved by the ethical committee of the University of Graz (GZ. 39/86/63 ex 2014/15). The overall theoretical framework of the project was the COM-B model [21]. COM-B stands for Capability (i.e., delivery of knowledge about health-enhancing PA during the health resort stay), Opportunity (i.e., access to an exercise programme), Motivation (i.e., development of skills to maintain the adopted PA behaviour), and Behaviour. 

Potential study participants were adults between 30 and 65 years from 11 regions in Styria. Those who were assigned to attend a residential stay in a health resort were invited to have their PA behaviour measured beforehand. Participants with a valid accelerometer measurement (at least four days for at least 10 h) received PA counselling during their health resort stay. Participants in the intervention group (IG) received, in addition, a coupon for 12 initial JACKPOT sessions in a sports club in their home region free of charge. This coupon would support the IG participant to start a supervised programme. Afterwards, participants were encouraged to continue the supervised exercise programme, but they were required to pay a membership fee of €80 per semester. The control group (CG) participants received a booklet about PA and health [22]. The primary outcome was the amount of PA per week, measured by accelerometer, 12 months after the first PA measurement. In this paper, we focus on the secondary outcome: the recruitment into and the attendance level of JACKPOT.

### 2.2. Recruitment of the Health Resorts and Participants

We invited all 67 health resorts in Austria who had a contract with the social insurance companies for the provision of residential stays for our target group and provided written information about the project. In addition, we also made phone contact with all the health resorts that agreed to join the project.

The study participants came from 11 defined regions in Styria. According to the ZIP code, participants from eight regions were allocated to the IG and participants from three regions to the CG. The higher proportion of IG regions was chosen because we plan to stratify the IG participants based on the attendance level in future analyses.

Participant recruitment took place from October 2015 to February 2017. Insurance companies contacted all adults from the 11 regions between 30 and 65 years who were selected for a residential stay. About three weeks before the start of the residential stay, the participants received a letter with the offer to measure their PA behaviour with an accelerometer before the stay (=measurement 1). Those who did not meet the optimal PA guidelines, i.e., those who performed <300 min/week of moderate-intensity PA, were invited to participate in the study.

### 2.3. Intervention in the Health Resorts

In addition to the usual programme during the health resort stay, the intervention (IG and CG) consisted of individualised PA counselling, taking the results of the accelerometer measurement into account. Furthermore, the IG participants received a standardised so-called “starter package”. The package included information about the health benefits of PA as well as a coupon for 12 regional JACKPOT sessions free of charge after the residential stay. The CG participants received a booklet about the health benefits of regular PA.

In order to facilitate the incorporation of the intervention into the daily routine at the health resorts, the delivery of the intervention elements was flexible: i.e., the members of the health resorts decided how the information was presented, who presented it, and when the different intervention elements took place. In addition, during the last days of the residential stay, research group members called the IG participants and encouraged them to start the JACKPOT programme after their return home.

### 2.4. Intervention in the Sports Clubs

In the eight intervention regions, at least one sports club provided JACKPOT sessions twice a week. The regional instructors were already active in the local sports clubs before they were recruited for the JACKPOT programme. The attendance of a two-day tailored further education training programme was mandatory, and the instructors were provided with a manual with 12 designed lectures as examples. The standardised exercise programme consists of three components: 40 min endurance training, 30 min strength training, and 20 min behaviour change counselling. However, in the manual, it is explained that the 40 min of endurance training and the 30 min of strength training each include 10 min of moving equipment back and forth [23]. The maximum number of people in a training group was restricted to 12 people.

### 2.5. Measurements

Together with the accelerometer (=measurement 1), all study participants received a questionnaire, which included questions about sex, age (years), weight (kg), height (cm), highest level of education, and self-perceived fitness compared to people of the same age and sex, rated on a five-point scale, with the answer categories: much fitter, fitter, equally fit, less fit, and much less fit. Body mass index (BMI) was computed in kg/m².

Between 9 and 10 months after the start of the project, one research group member conducted structured interviews in 21 health resorts about the implementation of the intervention. Health resorts with study participants and with a location which allowed the visit of several health resorts within one day were selected.

Furthermore, to assess whether the participants received the intervention during their health resort stay, research group members called the IG and CG participants and clarified whether they received the PA counselling, as well as the starter package and the booklet, respectively.

The attendance level is expressed as the number of attended sessions out of the 12 JACKPOT sessions, defining ≥75% as “good attendance” [24]. The documentation was completed by the exercise instructors, who filled in attendance records for every session and also indicated the reasons in the case of drop-out. The reasons for drop-out were grouped into similar categories and coded.

Participatory observation (*n* = 13) was applied to measure (stop watch) the duration of the three components of the JACKPOT session.

We used the PRECIS-2 toolkit [17] to assess how pragmatic the project was. We provide scores not only for the secondary outcome (recruitment into the programme and attendance level) but also for the primary outcome (PA per week), which is not the focus of this paper. The reason for this is that we wanted to take the whole project into account when rating the project’s practicability. The tool has nine domains: eligibility, recruitment, setting, organisation, flexibility: delivery, flexibility: adherence, follow-up, primary outcome, and primary analysis. All dimensions were rated by five members of the research group on a 1–5 scale, with 5 being the most pragmatic. If the scores differed among the assessors, the scores were discussed until all assessors agreed on a score. The scoring is based on the judgement about the ease with which the trial results can be applied in a similar situation/setting. A score of 5 means, therefore, that the new process could be easily implemented in a particular real-world context.

### 2.6. Statistical Analysis

Data up to the end of June 2017 were considered in the analyses. We examined the normal distribution of the metric variables with the Shapiro-Wilk test. For non-normal distributions, we report the median and the quartiles (25th; 75th).

To compare differences between groups, we used t-tests, Wilcoxon tests, or Chi^2^ tests, depending on the distribution and level of measurement. The significance level was set at *p* < 0.05 (two-sided test). All the statistical analyses were performed using IBM SPSS Statistics for Windows, Version 24.0. (IBM Corporation, Armonk, NY, USA). 

## 3. Results

### 3.1. Health Resorts

Of the 67 contacted health resorts, 51 (76.1%) agreed to become part of the project, three (4.5%) decided to participate later, and 13 (19.4%) did not respond to the invitation. In terms of numbers of beds, the health resorts that agreed to join the programme did not differ from those that did not participate. 

### 3.2. Study Participants

**Figure 1:**
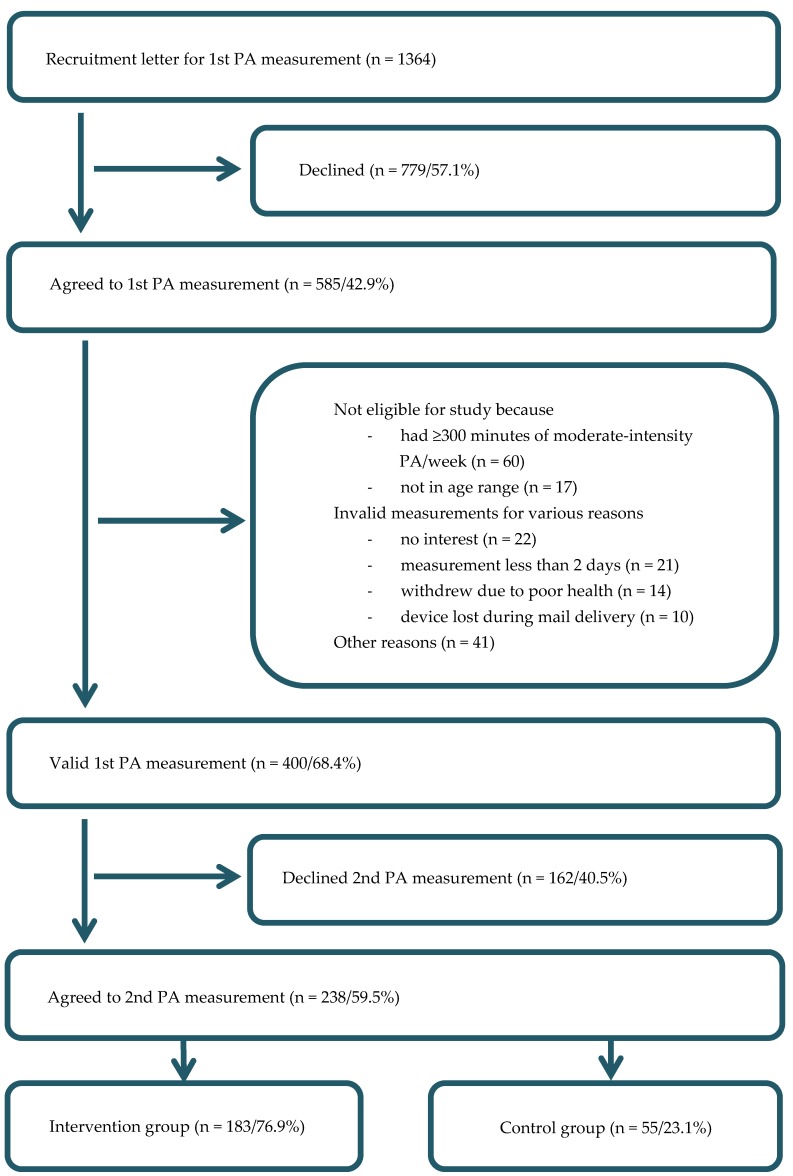
Overview of the recruitment flow. PA = physical activity.

The recruitment and process flow of the study participants is shown in Figure 1. Five hundred and eighty-five participants (IG = 445, CG = 140) agreed to wear an accelerometer for seven days and filled in the questionnaire. Due to age (<30 or >65 years), non-valid measurements, meeting the optimal PA recommendations, and other reasons, we collected 400 (68.4%) valid measurements (IG = 309, CG = 91). Of these, 238 (59.5%) participants agreed to take part in further measurements. The decliners did not differ from those who continued in terms of sex, BMI, education, or self-perceived fitness, but were older (median 55, Q_25_ = 50, Q_75_ = 59 years vs. median 53, Q_25_ = 49, Q_75_ = 57 years, *p* = 0.013).

The demographic characteristics of the study participants are shown in Table 1. There were no statistically significant differences between the IG and the CG.

### 3.3. Attendance Levels

Between October 2015 and June 2017, 90 (49%) of the 183 IG study participants who agreed to the 2nd PA measurement attended at least one JACKPOT session. In terms of sex, age, BMI, education, and self-perceived fitness level, there was no difference between those who attended at least one JACKPOT session (*n* = 90) and those who did not (*n* = 93).

The attendance levels for the JACKPOT programme are shown in Table 2. More than half of those who participated in at least one session had a good attendance level (≥75% of the 12 sessions).

Those with a good attendance level were older compared to those with <75% attendance (53.8 ± 6.3 years vs. 51.1 ± 6.2 years). Otherwise, no statistically significant differences were found (sex, BMI, education, and self-perceived fitness).

For 16 (39%) of the 41 JACKPOT participants who quit the programme before the 9th session, the reasons for the discontinuation were recorded by the instructors.

The most frequently mentioned reason was lack of time, followed by health reasons (Table 3).

### 3.4. Intervention Implementation

#### 3.4.1. Health Resorts

In the 21 health resorts, the counselling processes were differently organised in terms of who did the intervention, the time, when the intervention was delivered, and the context of the intervention. We did not find a preferred combination of the three intervention parameters among the 21 health resorts.

During the recruitment period (October 2015 to February 2017), 11 of the 51 health resorts did not accommodate or recruit any study participants from the 11 regions in Styria. Out of 39 health resorts, 17 (44%) delivered the starter package and the booklet, respectively, to at least 75% of the participants (IG and CG), 14 (36%) to at least 50% and 8 (20%) to 25% or less of the participants. We do not have records from one health resort.

#### 3.4.2. Sports Clubs

Out of 13 sports club instructors, 12 (92%) complied with the duration of 30 min endurance training (one almost with 29 min) and all instructors with the duration of 20 min strength training. However, only seven (54%) instructors dedicated time for reflection on how to successfully improve PA behaviour.

### 3.5. Practicability of the Project

In Table 4, the scores for the nine domains of the PRECIS-2 tool and their rationales are summarised.

In Table 4, it is shown that the basic procedures such as eligibility criteria, recruitment/first contact, setting, flexibility: delivery, and flexibility: attendance are “rather pragmatic” or “very pragmatic”. Only the organisation of the intervention is “indifferent pragmatic”. All the procedures that were related to the primary outcome of the scientific study were scored as “very explanatory”. The procedures needed for the secondary outcomes were scored between “indifferent pragmatic” and “rather pragmatic”.

## 4. Discussion

The aim of the health resort counselling was to make participants aware of their PA behaviour and to offer the IG participants a standardised sports club based PA programme (JACKPOT) in the community where they live. Almost half (49%) of the study participants from the IG attended JACKPOT sessions at least once. Of the attenders, 54% completed at least 75% of the 12 sessions.

In the recommendation statement by the U.S. Preventive Services Task Force [7], it was concluded that the studied PA messages increased aerobic activities to recommended levels. This conclusion was based on 88 trials with substantial variation in the mode of intervention (face-to-face or web-based), the duration of the delivery (ranging from a one-time mailing to monthly mailing over three years), and the applied behaviour change techniques. In our study, the counselling was face-to face, and was made up of PA counselling and, in the IG, the recommendation to attend a sports club based programme in the community.

During the one- to three-week health resort stay, different kinds of PA were performed and participants attended lectures about the benefits of different health behaviours and behaviour change techniques. However, according to Grossman and colleagues’ intensity classification [7], the participants experienced a low- (solely print material or ≤30 min of contact time) to medium-intensity (31 min to six hours of contact time) counselling intervention during their residential stay vs. high-intensity (>six hours of contact time) counselling intervention. The low to medium additional time needed for the counselling intervention was the reason for scoring the “organisation intervention” domain as “indifferent pragmatic”. 

The 49% recruitment rate into the JACKPOT programme is fairly consistent with findings from other studies recruiting people through the health care setting into a supervised exercise programme. For example, the recruitment of women with chronic disease risk factors into a three-month free-of-charge fitness centre prescribed by primary care providers was 40% [25]. A similar recruitment rate (41%) was achieved among overweight and obese patients for their participation in a standardised exercise programme referred by mainly outpatient departments [18]. In a systematic review with meta-analysis [24], the pooled level of recruitment through exercise referral schemes was 66% (95% CI 57% to 75%) across 14 observational studies. In this review, uptake was broadly defined in one of two ways: attendance at the initial consultation with an exercise professional or attendance at ≥1 supervised exercise sessions. The authors of this study therefore assumed that the higher recruitment rate compared to other studies is mainly based on the definition of successful recruitment also taking attendance at the initial consultation into account.

In accordance with Toobert et al. [26], we believe that our reasonable recruitment rate into the JACKPOT programme reflects the two-phase approach method employed (counselling and phone call). Although eligible IG participants were provided with all the necessary information about the study in their starter package, the pro-active call from a research group member was likely to help them take up the exercise programme.

As a “good attendance level” is differently specified in the studies, a comparison is difficult. In the successful Football Fans in Training (FFIT) study [27], 79% of the obese football fans attended six out of the 12 supervised sessions. In the study by Lackinger et al. [18], a good attendance level (>75% of all sessions) was achieved by 30% of the target group (non-communicable diseases such as obesity, type 2 diabetes or hypertension) after two months. Waterman et al. [25] defined “high participation” as at least 18 total class visits to a fitness centre over the 12-week trial period, but only 10% achieved the high participation categorisation in this study. In a systematic review about the effectiveness of exercise referral schemes, Williams et al. [28] reported that 12% to 42% of those who attended the first exercise session completed a full course. In our study, 69% of the study participants attended six out of the 12 sessions, 54% participants attended ≥75% of all the sessions, and 43% attended all the supervised sessions. In general, the attendance level in our study can be rated as “good” compared to the results of the above-mentioned single studies and the systematic review.

Because participants sometimes quit the JACKPOT programme without indicating the reason, we know the drop-out reasons from only around 40%. The listed reasons for drop-out are not surprising [29]. Most of the participants cite “lack of time” as the main reason for their drop-out. “Poor health status” is mentioned as the second most common reason. Some participants even indicate the specific illness (e.g., cruciate ligament rupture, disc prolapse (did not occur during JACKPOT), and cancer). Otherwise, it can only be speculated that these participants had a lack of self-efficacy to handle the 90 min PA, although JACKPOT was developed for unfit adults. Another speculation is that people did not find that the JACKPOT programme acted as a support to improve their well-being. In a study [30] of Indigenous Australian women who were recruited into a 12-week programme of exercise classes, the reasons for high and low attendance were investigated with semi-structured interviews. As in our study, personal health, logistics (e.g., distance), and lack of time due to competing obligations such as work and family were reported, but also instrumental support, which was not mentioned in our study, served as either a barrier or facilitator to regularly attend the programme.

With respect to the implementation of the standardisation of the interventions, we found limited studies to compare with our experience. The number of health resorts which delivered the starter package to at least 75% of the study participants was not satisfactory. This result shows how important it is to carry out the second-stage recruitment, i.e., phone call, to achieve a decent recruitment rate.

The proportion of instructors who implemented the standards for the two components of “endurance” and “strength training” was excellent. Reasons for this could be the two-day training with lectures and practical classes including communication training, as well as the existing experience of the instructors. However, only half of the instructors dedicated time to discussing behaviour change techniques derived from the Transtheoretical Model. In the 13 observed sessions, 32 (82%) of 39 key components were delivered. As a comparison, in the FFIT study [27], observers judged coaches to have delivered 81 of 93 (86%) key tasks in 26 delivery sessions. One reason for the disappointing adherence to discussing behaviour change techniques during the session could be the lack of detailed description in the manual and/or insufficient instruction during the two-day training programme. In the future one approach could be to settle behavior change frameworks (e.g., social ecological model) and behavior change techniques such as goal setting, feedback and monitoring, social support, identity, self-belief… [21] amongst the health resorts and the exercise instructors. This harmonisation would allow people to become more familiar with the behaviour change techniques. 

The use of the PRECIS-2 tool helped the research team to better understand how pragmatic the project was. It could be shown that we had few exclusion criteria (age and PA level), and therefore included a population that is similar to those who apply for a health resort stay. With respect to the basic processes (not taking the measurement of PA into account), we used recruitment approaches that built on existing routines. The delivery of the intervention was in line with existing care delivery processes in the health resorts. The project could also build on the good network of sports clubs all over Austria which could be used for the exercise intervention. However, funding was needed for the first 12 JACKPOT sessions, as well as the two-day training. It is too early to evaluate whether the fees to continue JACKPOT affected the adherence to the programme. There are indications that small fees do not affect financial accessibility [31].

When mainly focusing on the recruitment process into JACKPOT and the attendance level, the five assessors deemed that the activities could be “rather easily” implemented into a similar or same setting. For the organisation of the intervention, additional effort was needed from research group members. Taking the primary outcome, i.e., measurement of PA with accelerometer, into account, the project turns out to be “very explanatory”.

In terms of the external validity, we do not have data for those who declined the first PA measurement. We compared the participants with a valid first PA measurement who declined with those who agreed to continue the study and found that the decliners were older. It could be that the PA measurement presents a barrier for older participants. In addition, participants who were “good attenders” (≥75% of all classes) were older compared to participants with an attendance level <75%. As the age difference in both comparisons was not substantial (around two years), we assumed that the recruitment into the study and the attendance levels could be generalised to our settings.

This study has both strengths and limitations. Strengths include the satisfactory enrolment of the health resorts in Austria and the supportive involvement of insurance companies to recruit participants. Instead of relying on self-reporting, the instructors recorded the recruitment into the JACKPOT programme and the attendance level. We also documented why people dropped out. The comparison (sex, age, education, BMI, and self-rated fitness) of different groups, e.g., IG vs. CG, attenders vs. non-attenders, those who attended ≥75% of all sessions vs. those who attended less, allowed us to conclude that the results can be generalised and do not only apply to a convenience sample. We also documented and reported the implementation of the intervention, which has rarely been achieved.

This study also has limitations. How the intervention was performed in the health resort was systematically assessed in 21 (53%) of 40 health resorts with study participants. We therefore might have missed an opportunity to learn about intervention strategies that show a more successful recruitment level than the other strategies. Furthermore, participatory observation in the 13 sports clubs was conducted once per sports club, and was announced beforehand. This might not be enough to understand how consistently the instructors implemented the JACKPOT standards.

Other limitations were that, in most regions, only two JACKPOT sessions per week took place, which might have been, for some participants, on inconvenient days or at an inconvenient time. Two participants indicated that the JACKPOT location was too far away, although for 75% of the participants it took no more than 15 min to get to the sports club (results not shown). Finally, we recruited people from defined regions because the JACKPOT programme was only delivered in these regions. People from other regions could therefore not participate in JACKPOT.

## 5. Conclusions

The project is one of the first linking the health care sector with the widespread network of sports clubs in Europe. The process evaluation results showed that the project worked in a real-world setting, but also hints at the difficulties of implementing all the programme elements. We identified that the applied briefing provided to the many health resorts spread over Austria was not sufficient. Furthermore, exercise instructors should be more encouraged to include behavior change strategies in their sessions.

## Figures and Tables

**Table 1:** Characteristics of participants who agreed to the 2nd PA measurement (*n* = 238).

Participants’ Characteristics	Total (238)	IG (*n* = 183)	CG (*n* = 55)
Sex (%)			
Women	118 (49.6)	88 (48.1)	30 (54.5)
Men	120 (50.4)	95 (51.9)	25 (45.5)
Age in years (median and Q_25_; Q_75_)	53.0 (49.0; 57.0)	53.0 (48.0; 56.0)	54.0 (50.0; 59.0)
Missing	1		
Education (%)			
≤Completed apprenticeship	128 (54.5)	96 (53.3)	32 (58.2)
≥Intermed. voc. degree	107 (45.5)	84 (46.7)	23 (41.8)
Missing	3		
BMI (median and Q_25_; Q_75_)	26.2 (24.0; 29.7)	26.1 (24.0; 29.7)	26.2 (24.3; 29.7)
Missing	6		
Self-reported fitness level ^1^ (%)			
≥Fitter	86 (37.6)	67 (38.5)	19 (34.5)
Equally fit	93 (40.6)	69 (39.7)	24 (43.6)
≤Less fit	50 (21.8)	38 (21.8)	12 (21.8)
Missing	9		

Data are: number (%) or median (Q_25_–Q_75_). IG = intervention group, CG = control group. Intermed. voc. degree = intermediate vocational degree, BMI = Body mass index, ^1^ Question: How fit are you compared to people at your age and with your sex? Answer categories: much fitter, fitter, equally fit, less fit, much less fit.

**Table 2:** Attendance levels for the JACKPOT programme (*n* = 90).

Attended Sessions	*n*	*n* (Grouped)	%
1 session	9	23	25.6
2 sessions	5		
3 sessions	2		
4 sessions	7		
5 sessions	6	18	20.0
6 sessions	2		
7 sessions	3		
8 sessions	7		
9 sessions	6	49	54.4
10 sessions	3		
11 sessions	3		
12 sessions	37		

**Table 3:** Reasons for drop-out before the 75% attendance level (*n* = 16).

Stated Reasons	*n*	%
Lack of time	6	37.5
Poor health status of the participant	5	31.3
Prefers to continue on his/her own	2	12.5
Location too far away	1	6.3
Training too exhausting	1	6.3
Training not intensive enough	1	6.3

**Table 4:** PRECIS-2 scores, as rated by five members of the research group.

Domain	Score	Rationale
Eligibility criteria	5	All health resort patients from 11 regions in the state of Styria between 30 to 65 years were eligible. However, those meeting ≥300 min of moderate-intensity PA were excluded from the study.
Recruitment path	4	Health insurance companies sent an invitation letter as part of their routine communication and offered the PA measurement.
Setting	5	Health resorts which agreed to join the project did not differ from the health resorts which did not respond to the invitation or agreed to participate later. Therefore, the setting in our study is identical to the usual health resort setting. The JACKPOT programme takes place in already existing sports clubs. No additional equipment is needed.
Organisation of intervention	3	Health resort: The PA counselling as well as the delivery of the starter package were additional and new tasks for the health resort staff. Otherwise, no more staff and no additional training were required.
	3	Sports club: Exercise instructors who already worked in the sports clubs were recruited. The additional 2-day training for JACKPOT instructors and the first 12 sessions were funded.
Flexibility of experimental intervention: delivery	5	Health resorts: A framework for the content existed, but when, where, and by whom the intervention was delivered was flexible. Sports club session: The framework for each JACKPOT session was standardised and a manual with 12 lectures was delivered. However, instructors could adapt the content as long as they stuck to the framework.
Flexibility of experimental intervention: attendance	5	JACKPOT instructors actively encouraged participants to regularly attend JACKPOT sessions. Participants were not excluded based on their attendance level. However, the 12 sessions were only free for the first five months.
Follow-up	PO = 1	Primary Outcome: Delivery of the accelerometer devices was expensive and time consuming.
	SO = 3	Secondary Outcome: Participants’ attendance was recorded by attendance lists which are part of the instructors’ routine. In addition, we asked instructors to make notes about reasons for their drop-out and from which health resorts the participants came. To obtain the attendance lists from instructors required additional effort.
Relevance of primary and secondary outcome	PO = 5	Primary Outcome: Regular PA is an important outcome for the participants because regular PA reduces the risks of many non-communicable diseases.
	SO = 5	Secondary Outcome: Only if people continue with the newly adopted PA behaviour they will derive health benefits. Therefore, it is important to assess the success of the implementation, the recruitment of participants and the attendance level of the programme.
Analysis	PO = 1	Primary Outcome: Because of the three measurements, careful screening of the data and complex analyses to ensure the validity of the results will be required.
	SO = 4	Secondary Outcome: No special programmes or statistical skills, but regular records are required.

Score 1 = very explanatory, Score 2 = rather explanatory, Score 3 = indifferent pragmatic, Score 4 = rather pragmatic, Score 5 = very pragmatic. PO = primary outcome (minutes of moderate- to vigorous-intensity PA per week), SO = secondary outcome (recruitment into the programme and level of attendance).

## References

[1] Bamia C., Orfanos P., Juerges H., Schöttker B., Brenner H., Lorbeer R., Aadahl M., Matthews C.E., Klinaki E., Katsoulis M. (2017). Self-rated health and all-cause and cause-specific mortality of older adults: Individual data meta-analysis of prospective cohort studies in the CHANCES Consortium. Maturitas.

[2] Lee I.-M., Shiroma E.J., Lobelo F., Puska P., Blair S.N., Katzmarzyk P.T. (2012). Effect of physical inactivity on major non-communicable diseases worldwide: An analysis of burden of disease and life expectancy. Lancet.

[3] Shortreed S.M., Peeters A., Forbes A.B. (2013). Estimating the effect of long-term physical activity on cardiovascular disease and mortality: Evidence from the Framingham Heart Study. Heart.

[4] WHO (World Health Organization) Global Recommendations on Physical Activity for Health. http://www.who.int/dietphysicalactivity/factsheet_recommendations/en/.

[5] Statistik Austria Gesundheitsdeterminanten: Körperliche Aktivität in der Freizeit. http://www.statistik.at/web_de/statistiken/menschen_und_gesellschaft/gesundheit/gesundheitsdeterminanten/koerperliche_aktivitaet/index.html.

[6] Bull F.C., Gauvin L., Bauman A., Shilton T., Kohl H.W., Salmon A. (2010). The Toronto charter for physical activity: A global call for action. J. Phys. Act. Health.

[7] Grossman D.C., Bibbins-Domingo K., Curry S.J., Barry M.J., Davidson K.W., Doubeni C.A., Epling J.W., Kemper A.R., Krist A.H., Kurth A.E. (2017). Behavioral counseling to promote a healthful diet and physical activity for cardiovascular disease prevention in adults without cardiovascular risk factors: U.S. Preventive Services Task Force recommendation statement. JAMA.

[8] Bundesministerium für Frauen und Gesundheit Öffentliches Gesundheitsportal Österreichs: Kuraufenthalt. https://www.gesundheit.gv.at/gesundheitssystem/leistungen/antraege/kurantrag.

[9] Statistik Styria Email-Communication: Styrian Population in 2015. http://www.statistik.steiermark.at/cms/ziel/103034729/DE/URL.

[10] Pensionsversicherungsanstalt Pensionsversicherungsanstalt: Jahresbericht 2015. http://www.pensionsversicherung.at/cdscontent/load?contentid=10008.631742&version=1467614813.

[11] Hawley-Hague H., Horne M., Skelton D.A., Todd C. (2016). Review of how we should define (and measure) adherence in studies examining older adults’ participation in exercise classes. BMJ Open.

[12] Visek A.J., Olson E.A., DiPietro L. (2011). Factors predicting adherence to 9 months of supervised exercise in healthy older women. J. Phys. Act. Health.

[13] Withall J., Jago R., Fox K.R. (2012). The effect a of community-based social marketing campaign on recruitment and retention of low-income groups into physical activity programmes—A controlled before-and-after study. BMC Public Health.

[14] Zwarenstein M., Treweek S., Gagnier J.J., Altman D.G., Tunis S., Haynes B., Oxman A.D., Moher D. (2008). Improving the reporting of pragmatic trials: An extension of the CONSORT statement. BMJ.

[15] Loudon K., Zwarenstein M., Sullivan F.M., Donnan P.T., Gágyor I., Hobbelen H.J.S.M., Althabe F., Krishnan J.A., Treweek S. (2017). The PRECIS-2 tool has good interrater reliability and modest discriminant validity. J Clin. Epidemiol..

[16] Zwarenstein M., Treweek S., Loudon K. (2017). PRECIS-2 helps researchers design more applicable RCTs while CONSORT extension for pragmatic trials helps knowledge users decide whether to apply them. J. Clin. Epidemiol..

[17] Loudon K., Treweek S., Sullivan F., Donnan P., Thorpe K.E., Zwarenstein M. (2015). The PRECIS-2 tool: Designing trials that are fit for purpose. BMJ.

[18] Lackinger C., Wilfinger J., Mayerhofer J., Strehn A., Dick D., Dorner T.E. (2017). Adherence to and effects on physical function parameters of a community-based standardised exercise programme for overweight or obese patients carried out by local sports clubs. Public Health.

[19] Moore G.F., Audrey S., Barker M., Bond L., Bonell C., Hardeman W., Moore L., O’Cathain A., Tinati T., Wight D. (2015). Process evaluation of complex interventions: Medical Research Council guidance. BMJ.

[20] Lackinger C., Strehn A., Dorner T.E., Niebauer J., Titze S. (2015). Health resorts as gateways for regional, standardised, sports club based exercise programmes to increase the weekly time of moderate- to vigorous-intensity physical activity: Study protocol. BMC Public Health.

[21] Michie S., Atkins L., West R. (2014). The Behaviour Change Wheel—A Guide to Designing Interventions.

[22] Fonds Gesundes Österreich (2016). Bewegung. Gesundheit für Alle.

[23] Sozialversicherungsanstalt der Gewerblichen Wirtschaft JACKPOT: Manual Fuer Die JACKPOT-Bewegungseinheiten. http://jackpot.fit/data/jackpot_stundenbilder.pdf.

[24] Pavey T., Taylor A., Hillsdon M., Fox K., Campbell J., Foster C., Moxham T., Mutrie N., Searle J., Taylor R. (2012). Levels and predictors of exercise referral scheme uptake and adherence: A systematic review. JECH.

[25] Waterman M.R., Wiecha J.M., Manne J., Tringale S.M., Costa E., Wiecha J.L. (2014). Utilization of a free fitness center-based exercise referral program among women with chronic disease risk factors. J. Community Health.

[26] Toobert D.J., Strycker L.A., Glasgow R.E., Bagdade J.D. (2002). If you build it, will they come?. Patient Educ. Couns..

[27] Hunt K., Wyke S., Gray C.M., Anderson A.S., Brady A., Bunn C., Donnan P.T., Fenwick E., Grieve E., Leishman J. (2014). A gender-sensitised weight loss and healthy living programme for overweight and obese men delivered by Scottish Premier League football clubs (FFIT): A pragmatic randomised controlled trial. Lancet.

[28] Williams N.H., Hendry M., France B., Lewis R., Wilkinson C. (2007). Effectiveness of exercise-referral schemes to promote physical activity in adults: Systematic review. Br. J. Gen. Pract..

[29] Kelly S., Martin S., Kuhn I., Cowan A., Brayne C., Lafortune L. (2016). Barriers and facilitators to the uptake and maintenance of healthy behaviours by people at mid-life: A rapid systematic review. PLoS ONE.

[30] Canuto K.J., Spagnoletti B., McDermott R.A., Cargo M. (2013). Factors influencing attendance in a structured physical activity program for aboriginal and Torres Strait Islander women in an urban setting: A mixed methods process evaluation. Int. J. Equity Health.

[31] Arsenijevic J., Groot W. (2017). Physical activity on prescription schemes (PARS): Do programme characteristics influence effectiveness? Results of a systematic review and meta-analyses. BMJ Open.

